# Conhecimento sobre a Doença e a Prática de Atividade Física em Crianças e Adolescentes com Cardiopatia Congênita

**DOI:** 10.36660/abc.20180417

**Published:** 2020-05-22

**Authors:** Elisandra Furlan de Lima Campos, Lisiane Perin, Melina Assmann, Fernanda Lucchese, Lucia Campos Pellanda

**Affiliations:** 1 Programa de Pós-Graduação em Ciências da Saúde: Cardiologia Instituto de Cardiologia do Rio Grande do Sul Fundação Universitária de Cardiologia Porto AlegreRS Brasil Programa de Pós-Graduação em Ciências da Saúde: Cardiologia. Instituto de Cardiologia do Rio Grande do Sul – Fundação Universitária de Cardiologia, Porto Alegre, RS – Brasil; 2 Departamento de Saúde Coletiva Universidade Federal de Ciências da Saúde de Porto Alegre Porto AlegreRS Brasil Departamento de Saúde Coletiva – Universidade Federal de Ciências da Saúde de Porto Alegre, Porto Alegre, RS – Brasil

**Keywords:** Cardiopatias Congênita/fisiopatologia, Cianose, Criança, Adolescente, Sistema de Informação em Saúde, Atividade Física

## Abstract

**Fundamento:**

O conhecimento sobre a própria doença pode ser importante para o autocuidado em pacientes com vários problemas e abrange a informação sobre o diagnóstico até as implicações clínicas mais importantes.

**Objetivo:**

Identificar o nível de conhecimento de crianças e adolescentes com cardiopatia congênita (CC) sobre a sua doença, e analisar a relação entre o nível de conhecimento e a prática de atividade física.

**Métodos:**

Estudo transversal com 335 pacientes com CC, de 8 a 13 anos, acompanhados em um serviço de cardiologia pediátrica de referência no Sul do Brasil. Os pacientes foram entrevistados em relação ao seu conhecimento sobre a CC e foi realizada revisão dos prontuários para obtenção de detalhes sobre a cardiopatia e os procedimentos. Foi utilizado o nível de significância p < 0,05.

**Resultados:**

Mais de 50% das crianças e adolescentes não sabiam referir o nome de sua doença ou explicá-la. Após OR ajustado (OR_aj_), mostraram potencial para respostas incorretas ou não saber sua doença os pacientes cianóticos em relação aos acianóticos (OR_aj_: 2,29; IC95%: 1,76-6,71; p=0,019); crianças com menor nível de escolaridade (OR_aj_: 2,20; IC95%: 1,81-5,86; p=0,025); e não praticantes de atividade física (OR_aj_: 1,88; IC95%: 1,09-3,45; p=0,011).

**Conclusão:**

As crianças e adolescentes cianóticos, com menor nível de escolaridade e que não praticavam de atividade física apresentaram pouco conhecimento sobre a sua doença. Há necessidade do desenvolvimento de estratégias de intervenções educativas para aumento do conhecimento e mudança comportamental na promoção da atividade física, de acordo com a complexidade da CC. (Arq Bras Cardiol. 2020; 114(5):786-792)

## Introdução

O conhecimento da própria doença é um fator importante para o autocuidado em pacientes com cardiopatia congênita (CC)^1^ e abrange desde a informação sobre o diagnóstico até as implicações clínicas mais importantes.^2^ A CC é responsável por 0.8–1.2% de todos os defeitos congênitos e tem uma prevalência de cerca de 5,8 por 1.000 pessoas.^3^ A incidência de CC no Brasil é estimada em torno de 26.000 novos casos por ano.^4^

Para minimizar o risco de complicações e para melhorar o estado de saúde, espera-se que os pacientes adotem certos comportamentos de saúde, como práticas de atividade física, de alimentação saudável e higiene bucal.^5^ No entanto, a complexidade das cardiopatias e o conceito recorrente sobre a necessidade de restrição física geram dúvidas nos responsáveis e nos próprios profissionais de saúde a respeito dos níveis adequados de atividade física para crianças e adolescentes com CC.^6^ Além disto, as orientações se modificam ao longo do tempo, após correção da cardiopatia.^7^ Assim, muitas vezes a família ou o próprio paciente restringem as atividades físicas sem que isso represente uma orientação médica.

Poucos estudos foram realizados sobre o conhecimento específico de doenças como a CC em crianças, adolescentes ou adultos. Portanto, há lacunas de informações nas diferentes faixas etárias e a maioria dos estudos apresenta um pequeno número de pacientes capaz de proporcionar extrapolação de resultados.^5,8-11^ Portanto, identificar os níveis de conhecimento da criança com CC sobre a sua doença pode permitir o melhor planejamento de programas de educação em saúde que contribuam para minimizar as dúvidas em relação à prática de AF e melhorar a adesão ao tratamento. Assim, o propósito deste estudo foi identificar o nível de conhecimento de crianças e adolescentes com CC sobre a sua doença, e analisar a relação entre o nível de conhecimento e a prática de atividade física.

## Métodos

Trata-se de um estudo transversal que incluiu crianças e adolescentes com CC e idade entre 8 e 13 anos, em acompanhamento no Ambulatório Pediátrico do Instituto de Cardiologia do Rio Grande do Sul, realizado no período de fevereiro de 2017 a fevereiro de 2018. O protocolo do estudo foi aprovado pelo Comitê de Ética em Pesquisa da Fundação Universitária de e todos os participantes e responsáveis assinaram o Termo de Consentimento Livre e Esclarecido (TCLE) e Termo de Assentimento (TA).

**Figura 1:**
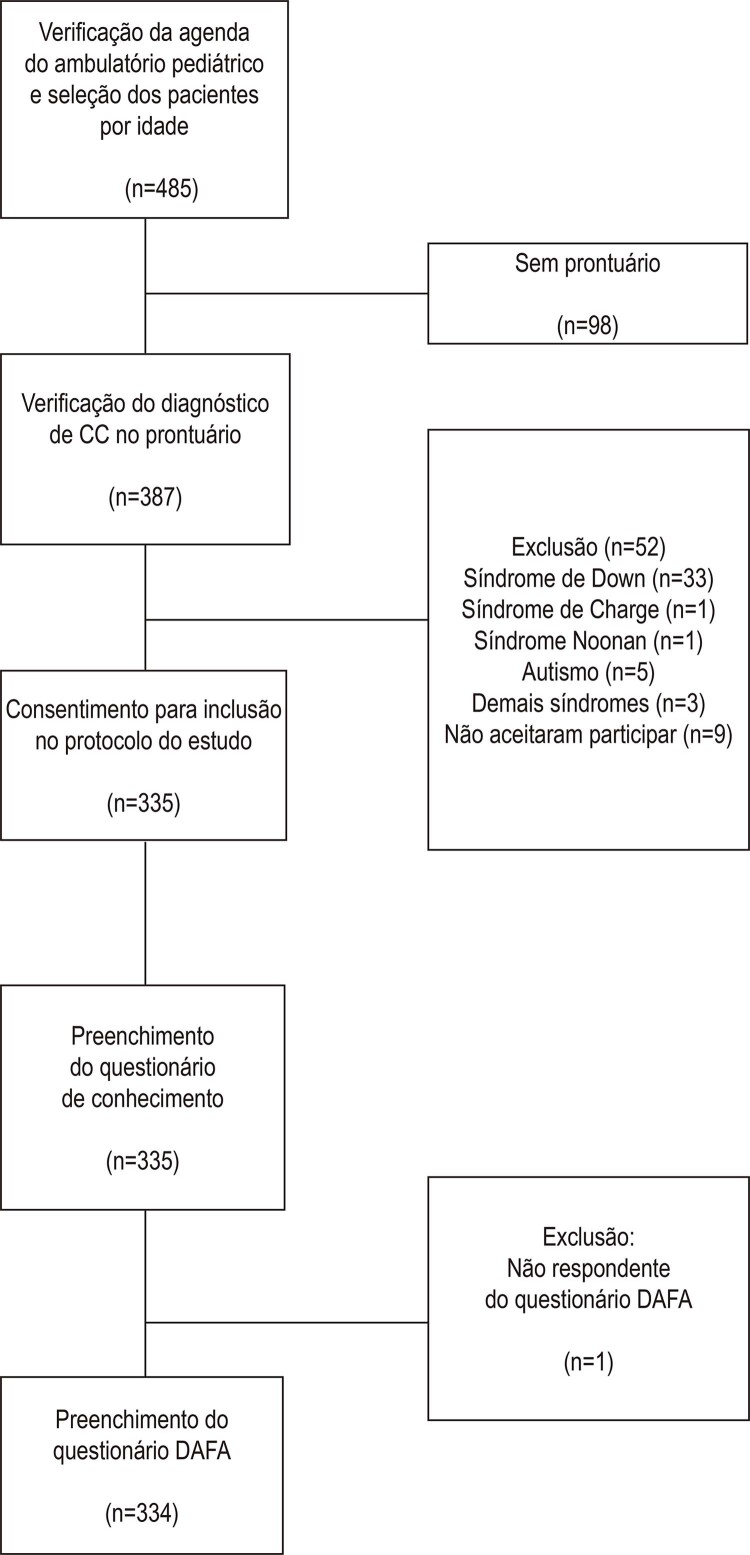
– *Fluxograma. CC: cardiopatia congênita; DAFA: Dia Típico de Atividade Física e Alimentar.*

Os pacientes foram incluídos de forma consecutiva durante um ano, a partir da lista de consultas agendadas durante o período do estudo. Foram excluídos os pacientes portadores de Síndrome de Down, Síndrome de Noonan, Síndrome de Charge, autismo, arritmias e síndromes que comprometem o entendimento sobre a sua doença. A faixa etária dos participantes foi verificada na agenda do ambulatório. Após a inclusão por idade, os respectivos prontuários foram analisados para confirmação da CC (Figura 1).

As entrevistas das crianças foram realizadas na sala de espera do ambulatório, onde foram explicados os objetivos e o protocolo do estudo para o paciente e para seu responsável. A coleta de dados foi realizada pelo mesmo entrevistador (EFLC), com vestimenta informal e teve duração entre 6 e 20 minutos.

Foi elaborado um questionário semiestruturado, baseado no questionário *Leuven Knowledge Questionnaire for Congenital Heart Disease* (LKQCHD)^6^sobre o conhecimento em CC. Os dados sociodemográficos e clínicos como internações prévias, procedimento hemodinâmico e cirúrgico foram extraídos do prontuário do paciente. As informações sobre a idade do diagnóstico da CC foram obtidas diretamente com os pais ou com os devidos responsáveis, de forma que as CCs foram classificadas como lesões mínimas (LM), acianóticas sem repercussão (ASR), acianóticas com repercussão (ACR) e cianóticas (CI).^11^ As crianças e adolescentes foram convidados a explicar, com suas próprias palavras, o que compreendiam sobre a sua doença. Foi realizado análise de conteúdo das respostas explicativas das crianças e adolescentes quanto ao conhecimento sobre a sua doença por dois médicos especialistas em cardiologia pediátrica (M.A. e L.C.P.) e, posteriormente, o nível de conhecimento foi classificado em 4 grupos: Correto (C), Parcialmente Correto (P/C), Incorreto (IN) e Não Sabe (NS).

Para avaliação do nível de atividade física foi utilizado parcialmente o instrumento Dia Típico de Atividade Física e Alimentar (DAFA). Utilizou-se a parte da atividade física que ilustra 11 tipos de atividades físicas em três intensidades distintas. O nível geral de atividade física foi determinado ao somar os escores das atividades que a avaliados referiam realizar na maioria dos dias da semana. Atribuiu-se três pesos distintos como forma de ponderar as atividades assinaladas por eles: peso um para atividades de intensidade leve, peso três para atividades de intensidade moderada e peso nove para atividades de intensidade vigorosa. O escore pode alcançar até 143 pontos, indicando as crianças menos ativas, intermediárias ou mais ativas.^12,13^ Tomando-se como base as separatrizes dos quartis [mediana de 25,0 (1º - 3º quartil: 16,0 – 36,0)] as pontuações do DAFA foram classificadas em três categorias: pontuações extremamente baixas DAFA ≤ 16,0, pontuações intermediárias em torno da mediana 16,0 < DAFA ≤ 36,0 e pontuações extremamente elevadas DAFA > 36,0.

O cálculo amostral foi realizado no programa WinPepi® versão 11.19^14^. Foi considerada a proporção de 50% de crianças com algum tipo de conhecimento sobre a sua doença, com poder estatístico de 90% e margem de erro 5%. Assim, a amostra foi estimada em 325 pacientes. No desenvolvimento do estudo, após a inclusão de 335 pacientes, identificou-se que os pacientes agendados já haviam sido avaliados e não havia novas inclusões no ambulatório.

### Análise estatística

As variáveis categóricas foram descritas sob a forma de números absolutos e percentagens, e as contínuas, sob a forma de médias e desvios padrão. Na distribuição das variáveis contínuas foi empregado o teste de Kolmogorov Smirnov, onde p>0,05 foi indicativo de dados simétricos.

Para identificar os fatores relacionados à prevalência do conhecimento IN/NS empregou-se uma análise bivariada pelo teste Qui-quadrado de Pearson complementado pela medida de efeito Odds Ratio (OR) bruto.^15^ Para verificar a existência de diferenças entre o conhecimento sobre CC nos diferentes níveis de atividade física, realizou-se o teste Análise de variância oneway com teste post hoc Sheffé.

Para avaliar a influência das variáveis estudadas sobre o nível de conhecimento IN/NS foi utilizado o modelo de regressão de Poisson. Na composição do modelo consideraram-se as variáveis que obtiveram significância ≤ 0,200 na análise bivariada não ajustada. Na análise ajustada foi utilizado o método Stepwise com eliminação retrógrada (backward). Permaneceram no modelo final apenas as variáveis associadas a um valor de p<0,05.^16^ Para critérios de decisão estatística adotou-se o nível de significância de 5%. Os testes foram realizados com o software *Statistical Package for Social Sciences* 20.0 (SPSS Inc., Chicago, IL, USA, 2011) para Windows.

## Resultados

Os resultados apresentados referem-se a uma amostra de 335 crianças com CC divididos em três grupos independentes, segundo o nível de conhecimento sobre a doença. A Tabela 1 apresenta a caracterização geral da amostra segundo a classificação para o conhecimento sobre a CC. Houve predomínio do sexo masculino (51,9%); idade 10 anos (21,2%), média de idade 10,5±1,68 anos; escolaridade entre 4º e 5º ano (40,6%); acianóticos com repercussão (55,5%); crianças que foram internadas (67,2%); crianças não tratadas com procedimento cirúrgico (60%) e crianças praticantes de AF (90,1%). O instrumento DAFA apresentou pontuações variando de 2,0 a 92,0 pontos, com média de 27,6 ± 14,2, mediana de 25,0 (1º - 3º quartil: 16,0 – 36,0) pontos. Considerando o nível de AF apontado pelo instrumento, verificou-se através dos quartis que os casos pouco ativos apresentaram pontuações DAFA ≤ 16,0 pontos, enquanto os muitos ativos apresentaram pontuações ≥ 36,0 pontos.

**Tabela 1:** – Caracterização geral da amostra segundo a classificação para o conhecimento

Variáveis	Total^**A**^ (n=335)		Conhecimento sobre cardiopatia congênita^**B**^						
			Correta/Parcial (n=148)		Incorreta (n=62)		Não sabe (n=125)		p*
	n	%	n	%	n	%	n	%	
**Sexo**									0,367*
Masculino	174	51,9	77	47,8	26	16,1	58	36,0	
Feminino	161	48,1	71	40,8	36	20,7	67	38,5	
**Idade**									0,033*
8	48	14,3	12	25,0	11	22,9	25	52,1	
9	60	17,9	27	45,0	9	15,0	24	40,0	
10	71	21,2	39	54,9	10	14,1	22	31,0	
11	48	14,3	20	41,7	06	12,5	22	45,8	
12	50	14,9	22	44,0	09	18,0	19	38,0	
13	58	17,3	28	48,3	17	29,3	13	22,4	
**Escolaridade (ano)**									0,009*
Pré-escola, 1º, 2º, 3º	89	26,6	27	30,3	17	19,1	45	50,6	
4º, 5º	136	40,6	63	46,3	23	16,9	50	36,8	
6º	43	12,8	22	51,2	8	18,6	13	30,2	
7º	48	14,3	29	60,4	8	16,7	11	22,9	
8º	19	5,7	7	36,8	6	31,6	6	31,6	
Classificação da CC									<0,001*
LM/ASR	81	24,2	37	45,7	11	13,6	33	40,7	
ACR	186	55,5	97	52,2	27	14,5	62	33,3	
CI	68	22,7	14	20,6	24	35,3	30	44,1	
**Internação**									0,044*
Sim	225	67,2	110	48,9	39	17,3	76	33,8	
Não	110	32,8	38	34,5	23	20,9	49	44,5	
**Procedimento cirúrgico**									0,015*
Sim	134	40	48	35,8	24	17,9	62	46,3	
Não	201	60	100	49,8	38	18,9	63	31,3	
**Prática de atividade física**									0,015*
Sim	302	90,1	141	46,7	53	17,2	109	36,1	
Não	33	9,9	7	21,2	10	30,3	16	48,5	
DAFA [Média±dp]	27,6±14,2		28,9±13,8		26,1±12,8		27,6±14,2		0,285^¥^

A: Percentuais obtidos sobre o total da amostra; B: Percentuais obtidos com base em cada categoria de respostas; *Teste Qui-Quadrado de Pearson; **Classificação das cardiopatias congênitas (CC): Lesões mínimas (LM); Acianóticos sem repercussão (ASR); Acianóticos com repercussão (cirurgia/hemodinâmica) (ACR); Cianóticos (CI); ¥: Análise de variância (One way) – Post hoc Sheffè.

No que se refere à comparação das variáveis do perfil das crianças em relação ao nível de conhecimento sobre CC, houve diferença significativa **e**ntre as faixas de idade (p=0,033), escolaridade (p=0,009), classificação da CC (p<0,001), internação (p= 0,044), procedimento cirúrgico (p= 0,015) e prática de AF (p= 0,015). Não houve diferença significativa (p=0,285) entre nível de AF avaliado pelo DAFA com conhecimento sobre CC.

De acordo com a Tabela 2, no que se refere ao OR ajustado, os maiores efeitos univariados indicaram que os pacientes com menor nível de escolaridade (pré-escola, 1º, 2º e 3º ano) apresentaram 2,20 (IC95%: 1,81-5,86) vezes mais chance de responder de forma incorreta ou não sabiam responder quando comparados aos pacientes com maior nível de escolaridade, 8º ano (p=0,025). Em relação à classificação da CC, os pacientes cianóticos apresentaram 2,29 (IC95%: 1,76-6,71) vezes mais chance de responder de forma incorreta ou não sabiam responder quando comparados aos pacientes acianóticos com repercussão (p=0,019). Quanto à prática de atividade física, os pacientes que não praticavam apresentaram 1,88 (IC95%: 1,09 3,34) vezes mais chance de responder incorreta ou não sabiam responder quando comparados aos pacientes que praticavam (p=0,011).

**Tabela 2:** – Prevalência para o conhecimento Incorreto/Não sabe, análise bruta e ajustada sobre as variáveis dependentes representativas no estudo

Variáveis	Conhecimento Incorreto/Não sabe^**C**^ (n=187)		*Odds ratio* ^**C**^		*Odds ratio*_**aj**_^**D**^	
	n	%	OR (IC95%)	p	OR_**aj**_ (IC95%)	p*
**Idade**				0,418		0,068
8	36	19,3	2,71(1,18 – 6,21)		2,84 (1,44-8,56)	
9	33	17,6	1,10 (0,54 –2,27)		1,22 (1,09-2,88)	
10	32	17,1	0,74 (0,37 –1,48)		0,98 (0,55-1,66)	
11	28	15,0	1,26 (0,59 –2,73)		1,17 (0,89-2,67)	
12	28	15,0	1,11 (0,52 –2,37)		1,09 (0,66-1,99)	
13	30	16,0	1,0		1,0	
**Escolaridade (ano)**				0,589		0,025
Pré-escola, 1º, 2º, 3º	62	33,2	1,34 (0,48 – 3,77)		2,20 (1,81 – 5,86)	
4º, 5º	73	39,0	0,78 (0,25 –1,82)		2,19 (1,60 – 6,21)	
6º	21	11,2	0,56 (0,18 –1,69)		1,69 (1,19 – 2,49)	
7º	19	10,2	0,38 (0,13 –1,14)		0,46 (0,14 – 1,55)	
8º	12	6,4	1,0		1,0	
**Classificação da CC**				0,026		
LM/ASR	44	23,5	1,0		1,0	0,019
ACR	89	47,6	0,77 (0,46 – 1,30)		0,63 (0,31- 1,27)	
CI	54	28,9	3,24 (1,56 – 6,75)		2,29 (1,76 - 6,71)	
**Crianças que foram internadas**				0,182		0,287
Sim	115	61,5	1,15 (0,92-1,49)		1,09 (0,64-1,36)	
Não	72	38,5	1,0		1,0	
**Crianças tratadas com procedimento cirúrgico**				0,013		0,355
Sim	86	46,0	1,0		1,0	
Não	101	54,0	1,77 (1,13-2,78)		1,22 (0,85 – 1,87)	
**Praticantes de atividade física**				0,009		0,011
Sim	162	86,6	1,0		1,0	
Não	26	13,9	2,20 (1,13-4,29)		1,88 (1,09 – 3,45)	

C: Odds ratio bruto para estimar o risco do conhecimento Incorreto/Não sabe em relação às categorias agrupadas conhecimento Incorreto/Não sabe; D: Odds ratio ajustado para estimar o risco do conhecimento Incorreto/Não sabe, em relação às categorias agrupadas conhecimento Incorreto/Não sabe ajustado para às variáveis presentes no modelo. Classificação das CC: Lesões mínimas (LM); Acianóticos sem repercussão (ASR); Acianóticos com repercussão (cirurgia/hemodinâmica) (ACR); Cianóticos (CI); *Teste Qui-Quadrado de Pearson.

## Discussão

O presente estudo destaca que a maioria das crianças e adolescentes com CC que participaram das entrevistas não souberam dizer o nome da sua doença e tampouco explicá-la com suas próprias palavras. Poucos estudos têm avaliado o nível de conhecimento com a classificação da cardiopatia ou prática de AF. Os estudos disponíveis na literatura científica são de difícil padronização por diversas questões metodológicas, incluindo a ausência de questionário validado para crianças.

Em um estudo descritivo, a maioria dos adolescentes (54%) não sabiam o nome do seu defeito cardíaco em comparação com a maioria dos seus pais (78%), que por sua vez sabiam corretamente o nome do defeito cardíaco do seu filho. Entretanto, apenas 24% dos adolescentes e 30% dos pais conseguiram localizar corretamente as lesões defeituosas em um diagrama do coração.^17^

Um estudo verificou que pacientes com CC leve tiveram mais respostas incorretas em um questionário de conhecimento sobre a sua doença, em comparação aos pacientes com CC moderada (p<0,001).^9^ Este achado difere ao encontrado no presente estudo, onde as crianças cianóticas responderam de forma incorreta em relação àquelas com lesões mínimas.

Uma possível explicação para isso é que os pacientes que pertenceram ao grupo de doença com malformação menos complexa e com mínima repercussão hemodinâmica, entenderiam e explicariam de forma mais fácil a sua doença em comparação aos pacientes com doenças cianóticas cujas explicações são mais complexas. Por sua vez, tipos específicos de CC têm sido associados a diferenças significativas no Quoficiente Intelectual (QI) médio.^18^ Crianças com lesões cianóticas tendem a ter QIs médios mais baixos do que crianças com CC acianótica,^19^ o que não foi avaliado nesse estudo.

Após a implementação de um programa estruturado de educação para adolescentes e adultos com CC, um estudo apontou que um escore total médio de conhecimento no grupo que recebeu intervenção educativa (57%) foi significativamente maior em relação ao grupo controle (43%) (p <0,001). No entanto, apenas 24 pacientes (11%) no grupo intervenção atingiram o objetivo proposto pelo programa educativo. Após o ajuste para a idade do paciente, nível de escolaridade e complexidade da doença, a análise de regressão linear multivariada mostrou que a oferta de educação estruturada de CC foi um determinante independente de níveis mais elevados de conhecimento (p<0,001). Desta forma, adolescentes e adultos com maior nível de escolaridade e maior complexidade da doença foram significativamente correlacionados com maior conhecimento sobre a sua doença (p<0,001).^20^

A prática de AF esteve associada ao maior conhecimento e, isto pode ter ocorrido em função das crianças gostarem de praticar AF e/ou os pais preocuparem-se e questionarem sobre os limites da AF à equipe médica. Além disso, as crianças também poderiam ter recebido mais informações sobre a condição do seu problema cardíaco. Outra interpretação alternativa foi que as crianças com maior conhecimento se sentiram mais seguras para praticar atividade física.

Nas Recomendações da Sociedade Europeia para crianças com CC, há previsão do encorajamento do paciente para a prática de AF e descrevendo as indicações e suas intensidades para cada tipo de lesão.^6^ Da mesma forma, uma pesquisa relata a capacidade do paciente localizar o defeito cardíaco em um diagrama e o conhecimento sobre restrições físicas foi fortemente correlacionada com o conhecimento sobre esportes, sendo que em ambos foram maiores nos pacientes do sexo masculino.^10^ Em contrapartida, outro estudo mostrou que 38% dos adolescentes e 52% dos pais sabiam sobre a CC e da liberação para participação de esportes competitivos.^17^

Níveis mais baixos de exercício físico têm sido associados com um aumento da incidência de incapacidades e doenças incluindo hipertensão, obesidade e diabetes. Ao passo que, altos níveis de exercício físico estão associados com maior aptidão musculoesquelética e menor risco de incapacidade física e desenvolvimento de doença.^21^ No entanto, no caso das cardiopatias congênitas, é importante considerar que há muita variabilidade no nível permitido de acordo com a doença, o tipo de correção e a presença de sequelas. A interação entre CC e fatores de risco cardiovascular adquiridos pode apresentar efeitos somatórios para o futuro. Há indícios que comorbidades adquiridas sejam provavelmente prejudiciais. É importante destacar que a modificação do conhecimento, comportamento e do estilo de vida bem como o tratamento correto deve começar de forma precoce com foco de atenção cardiovascular contínua.^22^

O estudo teve como limitação o possível viés de memória, que pode ter afetado a acurácia das respostas.

## Conclusão

As crianças e adolescentes cianóticos, com menor nível de escolaridade e que não praticavam atividade física apresentaram pouco conhecimento sobre a sua doença. Há necessidade do desenvolvimento de estratégias de intervenções educativas para aumento do conhecimento e mudança comportamental na promoção da atividade física, de acordo com a complexidade da CC.
