# Supplementary material for: Conhecimento sobre a Doença e a Prática de Atividade Física em Crianças e Adolescentes com Cardiopatia Congênita
Source: Arq Bras Cardiol. 2020 May 22;114(5):786–92. [Article in Portuguese] doi: 10.36660/abc.20180417 (PMC8387008; doi:10.36660/abc.20180417)
Supplement: Translation

# Translation: Knowledge about the Disease and the Practice of Physical Activity in Children and Adolescents with Congenital Heart Disease

**Authors:** Elisandra Furlan de Lima Campos, Lisiane Perin, Melina Assmann, Fernanda Lucchese, Lucia Campos Pellanda

**Affiliations:** 1 Brazil Programa de Pós-Graduação em Ciências da Saúde: Cardiologia. Instituto de Cardiologia do Rio Grande do Sul – Fundação Universitária de Cardiologia, Porto Alegre, RS – Brazil; 2 Brazil Departamento de Saúde Coletiva – Universidade Federal de Ciências da Saúde de Porto Alegre, Porto Alegre, RS – Brazil

**Keywords:** Heart Defects, Congenital/physiopathology, Cyanosis, Child, Adolescents, Health Information Systems, Physical Activity

## Abstract

**Background:**

Knowledge about the disease itself can be important for self-care in patients with several problems and comprehends information about the diagnosis up to the most important clinical implications.

**Objective:**

To identify the level of knowledge of children and adolescents with congenital heart disease (CHD) about their illness, and to analyze the association between the level of knowledge and the practice of physical activity.

**Methods:**

Cross-sectional study with 335 patients with CHD, aged 8 to 13 years, followed at a referral pediatric cardiology service in Southern Brazil. Patients were interviewed regarding their knowledge about CHD and a review of medical records was performed to obtain details on heart disease and procedures. A significance level p<0.05 was used.

**Results:**

More than 50% of the children and adolescents did not know how to say the name of their disease or explain it. After adjusted OR (AOR), cyanotic patients in comparison to acyanotic ones (AOR: 2.29; 95%CI: 1.76-6.71; p = 0.019); children with lower level of schooling (AOR: 2.20; 95%CI: 1.81-5.86; p = 0.025); and those who did not practice physical activity (AOR: 1.88; 95%CI: 1.09-3.45; p = 0.011) showed potential for incorrect answers or did not know their disease.

**Conclusion:**

Cyanotic children and adolescents, with a lower level of schooling and who did not practice physical activity, had little knowledge about their disease. It is necessary to develop educational intervention strategies to increase knowledge and change behavior in physical activity promotion, according to the CHD complexity. (Arq Bras Cardiol. 2020; 114(5):786-792)

## Introduction

Knowledge of the disease itself is an important factor for self-care in patients with congenital heart disease (CHD)^1^ and ranges from information about the diagnosis to the most important clinical implications.^2^ CHD is responsible for 0.8–1.2% of all congenital defects and has a prevalence of approximately 5.8 per 1,000 individuals.^3^ The incidence of CHD in Brazil is estimated at around 26,000 new cases per year.^4^

To minimize the risk of complications and improve health status, patients are expected to adopt certain health behaviors, such as physical activity, healthy eating and oral hygiene practices.^5^ However, the complexity of heart diseases and the recurring concept of the need for physical restriction generate doubts among parents and health professionals about the adequate levels of physical activity for children and adolescents with CHD.^6^ Moreover, the guidelines change over time, after the heart disease repair.^7^ Therefore, often the family or the patients themselves restrict physical activities without this representing medical advice.

Few studies have been performed on the specific knowledge of diseases such as CHD in children, adolescents or adults. Therefore, there are information gaps in different age groups and most studies have a small number of patients that allows the extrapolation of results.^5,8-11^ Therefore, identifying the levels of the knowledge of a child with CHD about their disease can allow better planning of health education programs that will contribute to minimize doubts regarding the practice of physical activity (PA) and improve adherence to treatment. Thus, the aim of this study was to identify the level of knowledge of children and adolescents with CHD about their disease, and to analyze the association between the level of knowledge and the practice of physical activity.

## Methods

This is a cross-sectional study that included children and adolescents with CHD, aged between 8 and 13 years, followed at the Pediatric Outpatient Clinic of Instituto de Cardiologia do Rio Grande do Sul, carried out from February 2017 to February 2018. The study protocol was approved by the Research Ethics Committee of Fundação Universitária, and all participants and parents/guardians signed the Free and Informed Consent (FIC) form and Term of Assent (TA).

Patients were included consecutively for one year, from the list of medical appointments scheduled during the study period. Patients with Down Syndrome, Noonan Syndrome, Charge Syndrome, autism, arrhythmias and syndromes that compromise the understanding of their disease were excluded. The age range of the participants was checked on the outpatient clinic agenda. After inclusion by age, the respective medical records were analyzed to confirm the presence of CHD (Figure 1).

**Figure 1:** – *Flowchart. CHD: congenital heart disease; DAFA: Typical Physical Activity and Food Intake Day.*

The children’s interviews were carried out in the waiting room of the outpatient clinic, where the objectives and study protocol were explained to the patients and their parents/guardians. Data collection was carried out by the same interviewer (EFLC), who was informally dressed, and lasted between 6 and 20 minutes.

A semi-structured questionnaire was developed, based on the Leuven Knowledge Questionnaire for Congenital Heart Disease (LKQCHD)^6^ about knowledge of CHD. Sociodemographic and clinical data, such as previous hospitalizations, hemodynamic and surgical procedures, were extracted from the patient’s medical record. Information on the age at which the diagnosis of CHD was obtained was obtained directly from the parents or guardians, so that the CHDs were classified as minimal lesions (ML), acyanotic without implications (ASI), acyanotic with implications (AWI) and cyanotic (CY).^11^ The children and adolescents were asked to explain, in their own words, what they understood about their disease. The content analysis of the explanatory responses of children and adolescents regarding the knowledge of their disease was carried out by two physicians specialized in pediatric cardiology (M.A. and L.C.P.) and, subsequently, the level of knowledge was classified into 4 groups: Correct (C), Partially Correct (P/C), Incorrect (IN) and Doesn’t Know (DK).

To assess the level of physical activity, the Typical Physical Activity and Food Intake Day (DAFA) instrument was partially used. We used the part of physical activity that illustrates 11 types of physical activities in three different intensities. The overall level of physical activity was determined by adding the scores of the activities that the assessed individuals reported performing on most days of the week. Three different weights were assigned aiming to weigh the activities indicated by the patients: weight one for light intensity activities, weight three for moderate intensity activities and weight nine for vigorous intensity activities. The score can reach up to 143 points, indicating children who are less active, intermediate or more active.^12,13^ Based on quartiles limits [median of 25.0 (1^st^ - 3^rd^ quartile: 16.0 - 36.0)], the DAFA scores were classified into three categories: extremely low DAFA, scores ≤ 16.0, intermediate scores, around the median 16.0<DAFA≤ 36.0 and extremely high scores, DAFA>36.0.

The sample calculation was performed using the WinPepi® program version 11.1914. The proportion of 50% of children with some type of knowledge about their disease was considered, with a statistical power of 90% and 5% margin of error. Therefore, the sample was estimated at 325 patients. During the study development, after the inclusion of 335 patients, it was verified that the scheduled patients had already been evaluated and there were no new inclusions in the outpatient clinic.

### Statistical analysis

Categorical variables were described as absolute numbers and percentages, and the continuous variables as means and standard deviations. The Kolmogorov-Smirnov test was used in the distribution of continuous variables, where p >0.05 indicated symmetric data.

To identify the factors related to the prevalence of IN/DK knowledge, a bivariate analysis was used with Pearson's Chi-square test complemented by the measure of crude Odds Ratio (OR) effect.^15^ To verify the existence of differences between knowledge about CHD at different levels of physical activity, the One-way Analysis of Variance test with Sheffé *post hoc* test was performed.

To assess the influence of the studied variables on the level of knowledge IN/DK, the Poisson regression model was used. In the composition of the model, the variables that obtained significance ≤ 0.200 in the unadjusted bivariate analysis were considered. In the adjusted analysis, the backward-stepwise method was used. Only variables associated with a p value <0.05^16^ remained in the final model. A significance level of 5% was adopted for statistical decision criteria. The tests were performed with the software Statistical Package for Social Sciences 20.0 (SPSS Inc., Chicago, IL, USA, 2011) for Windows.

## Results

The results presented herein are related to a sample of 335 children with CHD divided into three independent groups, according to the level of knowledge about the disease. Table 1 shows the overall sample characterization according to the classification about CHD knowledge. There was a predominance of the male gender (51.9%); age 10 years (21.2%), mean age of 10.5 ± 1.68 years; schooling between 4^th^ and 5^th^ grades (40.6%); acyanotic individuals with implications (55.5%); children who were hospitalized (67.2%); children not treated with surgical procedure (60%) and children practicing PA (90.1%). The DAFA instrument showed scores ranging from 2.0 to 92.0 points, with an average of 27.6 ± 14.2, median of 25.0 (1^st^ - 3^rd^ quartile: 16.0 - 36.0) points. Considering the PA level indicated by the instrument, it was verified through the quartiles that the low active cases had DAFA scores ≤ 16.0 points, while the highly active ones had scores ≥ 36.0 points.

**Table 1:** – Overall characterization of the sample according to the classification for knowledge

Variables	Total^**A**^ (n=335)		Knowledge about congenital heart disease^**B**^						
			Correct / Partial (n=148)		Incorrect (n=62)		Does not know (n=125)		p*
	n	%	n	%	n	%	n	%	
**Gender**									0.367*
Male	174	51.9	77	47.8	26	16.1	58	36.0	
Female	161	48.1	71	40.8	36	20.7	67	38.5	
**Age**									0.033*
8	48	14.3	12	25.0	11	22.9	25	52.1	
9	60	17.9	27	45.0	9	15.0	24	40.0	
10	71	21.2	39	54.9	10	14.1	22	31.0	
11	48	14.3	20	41.7	06	12.5	22	45.8	
12	50	14.9	22	44.0	09	18.0	19	38.0	
13	58	17.3	28	48.3	17	29.3	13	22.4	
**Level of schooling (year)**									0.009*
Preschool, 1^st^, 2^nd^, 3^rd^	89	26.6	27	30.3	17	19.1	45	50.6	
4^th^, 5^th^	136	40.6	63	46.3	23	16.9	50	36.8	
6^th^	43	12.8	22	51.2	8	18.6	13	30.2	
7^th^	48	14.3	29	60.4	8	16.7	11	22.9	
8^th^	19	5.7	7	36.8	6	31.6	6	31.6	
CHD classification									<0.001*
ML/ASI	81	24.2	37	45.7	11	13.6	33	40.7	
AWI	186	55.5	97	52.2	27	14.5	62	33.3	
CY	68	22.7	14	20.6	24	35.3	30	44.1	
**Hospitalization**									0.044*
Yes	225	67.2	110	48.9	39	17.3	76	33.8	
No	110	32.8	38	34.5	23	20.9	49	44.5	
**Surgical procedure**									0.015*
Yes	134	40	48	35.8	24	17.9	62	46.3	
No	201	60	100	49.8	38	18.9	63	31.3	
**Physical activity**									0.015*
Yes	302	90.1	141	46.7	53	17.2	109	36.1	
No	33	9.9	7	21.2	10	30.3	16	48.5	
DAFA [Mean±SD]	27.6±14.2		28.9±13.8		26.1±12.8		27.6±14.2		0.285^¥^

A: Percentages obtained for the total sample; B: Percentages obtained based on each category of responses; * Pearson’s Chi-square test; ** Classification of CHD: Minimal lesions (ML); Acyanotic without implications (ASI); acyanotic with implications (AWI) (surgery/hemodynamics); Cyanotic (CY); ¥: Analysis of variance (One-way) - Sheffè Post-hoc test.

Regarding the comparison of the children's profile variables in relation to the knowledge level about CHD, there was a significant difference between the age groups (p = 0.033), level of schooling (p = 0.009), CHD classification (p<0.001), hospitalization (p = 0.044), surgical procedure (p = 0.015) and PA practice (p = 0.015). There was no significant difference (p = 0.285) between the level of PA assessed by DAFA with knowledge about CHD.

According to Table 2, regarding the adjusted OR, the greatest univariate effects indicated that patients with a lower level of schooling (pre-school, 1^st^, 2^nd^and 3^rd^ grades) were 2.20 (95% CI: 1, 81-5.86) times more likely to answer incorrectly or did not know how to answer when compared to patients with a higher level of schooling, 8^th^ grade (p = 0.025). Regarding the classification of CHD, cyanotic patients were 2.29 (95% CI: 1.76-6.71) times more likely to answer incorrectly or did not know how to answer when compared to acyanotic patients with implications (p = 0.019). As for the practice of physical activity, patients who did not practice were 1.88 (95% CI: 1.09 3.34) times more likely to answer incorrectly or did not know how to answer when compared to patients who practiced PA (p = 0.011).

**Table 2:** – Prevalence for Incorrect/Doesn’t know knowledge of disease, crude and adjusted analysis on representative dependent variables in the study

Variables	Incorrect / Doesn’t Know Knowledge (n=187)		*Odds ratio* ^**C**^		*Adjusted Odds ratio* ^***D***^	
	n	%	OR (IC95%)	p	_**adj**_OR (IC95%)	p*
**Age**				0.418		0.068
8	36	19.3	2.71(1.18 – 6.21)		2.84 (1.44-8.56)	
9	33	17.6	1.10 (0.54 –2.27)		1.22 (1.09-2.88)	
10	32	17.1	0.74 (0.37 –1.48)		0.98 (0.55-1.66)	
11	28	15.0	1.26 (0.59 –2.73)		1.17 (0.89-2.67)	
12	28	15.0	1.11 (0.52 –2.37)		1.09 (0.66-1.99)	
13	30	16.0	1.0		1.0	
**Level of schooling (year)**				0.589		0.025
Preschool, 1^st^, 2^nd^, 3^rd^	62	33.2	1.34 (0.48 – 3.77)		2.20 (1.81 – 5.86)	
4^th^, 5^th^	73	39.0	0.78 (0.25 –1.82)		2.19 (1.60 – 6.21)	
6^th^	21	11.2	0.56 (0.18 –1.69)		1.69 (1.19 – 2.49)	
7^th^	19	10.2	0.38 (0.13 –1.14)		0.46 (0.14 – 1.55)	
8^th^	12	6.4	1.0		1.0	
**CHD classification**				0.026		
ML/ASI	44	23.5	1.0		1.0	0.019
AWI	89	47.6	0.77 (0.46 – 1.30)		0.63 (0.31- 1.27)	
CY	54	28.9	3.24 (1.56 – 6.75)		2.29 (1.76 - 6.71)	
**Children who were hospitalized**				0.182		0.287
Yes	115	61.5	1.15 (0.92-1.49)		1.09 (0.64-1.36)	
No	72	38.5	1.0		1.0	
**Children treated with surgical procedure**				0.013		0.355
Yes	86	46.0	1.0		1.0	
No	101	54.0	1.77 (1.13-2.78)		1.22 (0.85 – 1.87)	
**Practiced physical activity**				0.009		0.011
Yes	162	86.6	1.0		1.0	
No	26	13.9	2.20 (1.13-4.29)		1.88 (1.09 – 3.45)	

C: crude odds ratio to estimate the risk of Incorrect/Doesn’t know knowledge in relation to the grouped categories Incorrect/Doesn’t know knowledge. D: Adjusted Odds ratio to estimate the risk of Incorrect/Doesn’t know knowledge, in relation to the grouped categories Incorrect/Don’t know knowledge adjusted for the variables present in the model. Classification of CHD: Minimal lesions (ML); acyanotic without implications (ASI); acyanotic with implications (AWI) (surgery/hemodynamics); Cyanotic (CY). * Pearson’s Chi-square test

## Discussion

The present study highlights the fact that most children and adolescents with CHD who participated in the interviews did not know how to say the name of their disease or explain it in their own words. Few studies have assessed the level of knowledge with the classification of heart disease or PA practice. The studies available in the scientific literature are difficult to standardize due to several methodological issues, including the absence of a validated questionnaire for children.

In a descriptive study, most adolescents (54%) did not know the name of their heart defect compared to most of their parents (78%), who in turn knew the name of their child's heart defect correctly. However, only 24% of adolescents and 30% of parents were able to correctly locate the defective lesions on a heart diagram.^17^

A study found that patients with mild CHD had more incorrect answers in a questionnaire about their disease, compared to patients with moderate CHD (p <0.001).^9^ This finding differs from that found in the present study, in which cyanotic children answered incorrectly in relation to those with minimal lesions.

A possible explanation for this is that patients who belonged to the disease group with less complex malformation and with minimal hemodynamic implications, would understand and explain their disease more easily, when compared to patients with cyanotic diseases, of which explanations are more complex. In turn, specific types of CHD have been associated with significant differences in the average Intellectual Quotient (IQ).^18^ Children with cyanotic disease tend to have lower average IQs than children with acyanotic CHD,^19^ which was not assessed in this study.

After the implementation of a structured education program for adolescents and adults with CHD, a study found that an average total score of knowledge in the group that received educational intervention (57%) was significantly higher compared to the control group (43%) (p<0.001). However, only 24 patients (11%) in the intervention group achieved the objective proposed by the educational program. After adjusting for the patient’s age, level of schooling and disease complexity, the multivariate linear regression analysis showed that the provision of structured education for CHD was an independent determinant of higher levels of knowledge (p <0.001). Therefore, adolescents and adults with a higher level of schooling and higher disease complexity were significantly correlated with greater knowledge about their disease (p <0.001).^20^

The practice of PA was associated with greater knowledge, and this may have occurred because children like to practice PA and/or parents are concerned about and questioned the limits of PA to the medical team. Moreover, children could also have received more information about the condition of their heart problem. Another alternative interpretation was that the children with more knowledge felt safer to practice physical activity.

In the European Society Recommendations for children with CHD, there is provision for encouraging the patient to practice PA and describing the indications and their intensities for each type of lesion.^6^ Likewise, a study reports that the patient’s ability to locate the heart defect on a diagram and knowledge about physical restrictions were strongly correlated with knowledge about sports, both of which were higher in male patients.^10^ In contrast, another study showed that 38% of adolescents and 52% of parents knew about CHD and the endorsement to participate in competitive sports.^17^

Lower levels of physical exercise have been associated with an increased incidence of disabilities and diseases, including hypertension, obesity and diabetes. In contrast, high levels of physical exercise are associated with greater musculoskeletal fitness and less risk of physical disability and development of diseases.^21^ However, in cases of CHD, it is important to consider that there is a lot of variability regarding the level of PA allowed according to the disease, the type of correction and the presence of sequelae. The interaction between CHD and acquired cardiovascular risk factors can have summing effects for the future. There are indications that acquired comorbidities are likely to be harmful. It is important to emphasize that the modification of knowledge, behavior and lifestyle, as well as the correct treatment, should start early with a focus on continuous cardiovascular care.^22^

The study had the possible memory bias as a limitation, which may have affected the accuracy of the answers.

## Conclusion

Cyanotic children and adolescents, with a lower level of education and who did not practice physical activity, had little knowledge about their disease. It is necessary to develop educational intervention strategies to increase knowledge and change behaviors regarding physical activity promotion, according to the CHD complexity.
